# Chlamydia and Perihepatitis (Fitz-Hugh-Curtis Syndrome) in an 18-Year-Old Male: A Case Report

**DOI:** 10.7759/cureus.86282

**Published:** 2025-06-18

**Authors:** Anup H Kotadia, Kenneth Raney

**Affiliations:** 1 Internal Medicine, Methodist Health System, Dallas, USA

**Keywords:** chlamydia trachomatis, fitz-hugh-curtis syndrome, male, perihepatitis, peritonitis

## Abstract

Perihepatitis, a complication of pelvic inflammatory disease, is most commonly observed in women. This condition results from inflammation of the liver capsule, leading to scarring and adhesions in the abdominal cavity with associated complications. We present the case of an 18-year-old male with three months of abdominal pain. Upon admission, he was diagnosed with peritonitis, and imaging revealed extraluminal air in the liver vasculature. He was found to be positive for *Chlamydia trachomatis*, leading to a diagnosis of chlamydia and perihepatitis. Recognizing pelvic inflammatory disease-like intraabdominal infections in male patients is crucial, given it has the potential to cause lifelong intraabdominal complications.

## Introduction

Perihepatitis (alternatively, Fitz-Hugh-Curtis syndrome) is a complication of pelvic inflammatory disease (PID). It is usually seen in females, although rare cases in males have been reported [1]. When discussing PID in male patients, it is better described as PID-like intra-abdominal infection, given the traditional ascending infectious route for PID is absent in males, and PID-like intra-abdominal infection will be assumed to be used for all further mentions of PID in males in this article. In women, 5% to 15% of PID cases develop perihepatitis [2]. The first reported cases in females were documented in the 1930s, but the first case reported in a male patient was not until 1970 [3]. The usual cause of PID is infection from either *Chlamydia trachomatis* or *Neisseria gonorrhoeae* [1], which causes inflammation of the liver capsule and parietal peritoneum of the abdominal wall [4]. The liver parenchyma is not involved [1]. *Chlamydia trachomatis* is responsible for approximately five times more cases of perihepatitis than *Neisseria gonorrhoeae* [3].

Patients with perihepatitis usually present with upper quadrant pain in the absence of gallstones and are often misdiagnosed with biliary colic or cholecystitis [1,5]. The gold standard for diagnosis is diagnostic laparoscopy, which shows adhesions between the liver capsule and abdominal wall in a characteristic violin string appearance [4]. However, due to its invasiveness, laparoscopy is rarely done unless it impacts management [2]. Perihepatitis typically responds well to antibiotics, but a common complication can be infertility and chronic pelvic pain.

Here, we report the case of an 18-year-old male with abdominal pain diagnosed with perihepatitis linked to a *Chlamydia trachomatis* infection.

## Case presentation

An 18-year-old male with no prior medical or surgical history presented after several hours of severe epigastric and right upper quadrant pain accompanied by fever. Upon further inquiry, the patient reported three months of intermittent abdominal pain, watery diarrhea, fever, and chills. His sexual history revealed female partners with condom use. On initial examination, the patient was afebrile, had a pulse of 94 bpm, was hypertensive at 147/84 mmHg, and had generalized abdominal tenderness to palpation without distention, rebound, or guarding. An admission complete blood count showed a white blood cell count of 3.9 x 109/L, hemoglobin of 15.3 g/dL, hematocrit of 44.8%, and platelets of 317 x 109/L. Liver chemistries were as follows: aspartate transaminase of 27 IU/L, alanine transaminase of 26 IU/L, alkaline phosphatase of 85 IU/L, total bilirubin of 1.3 μmol/L, and albumin of 4.2 g/dL. Admission lactic acid was 1.8 mmol/L.

**Figure 1:**
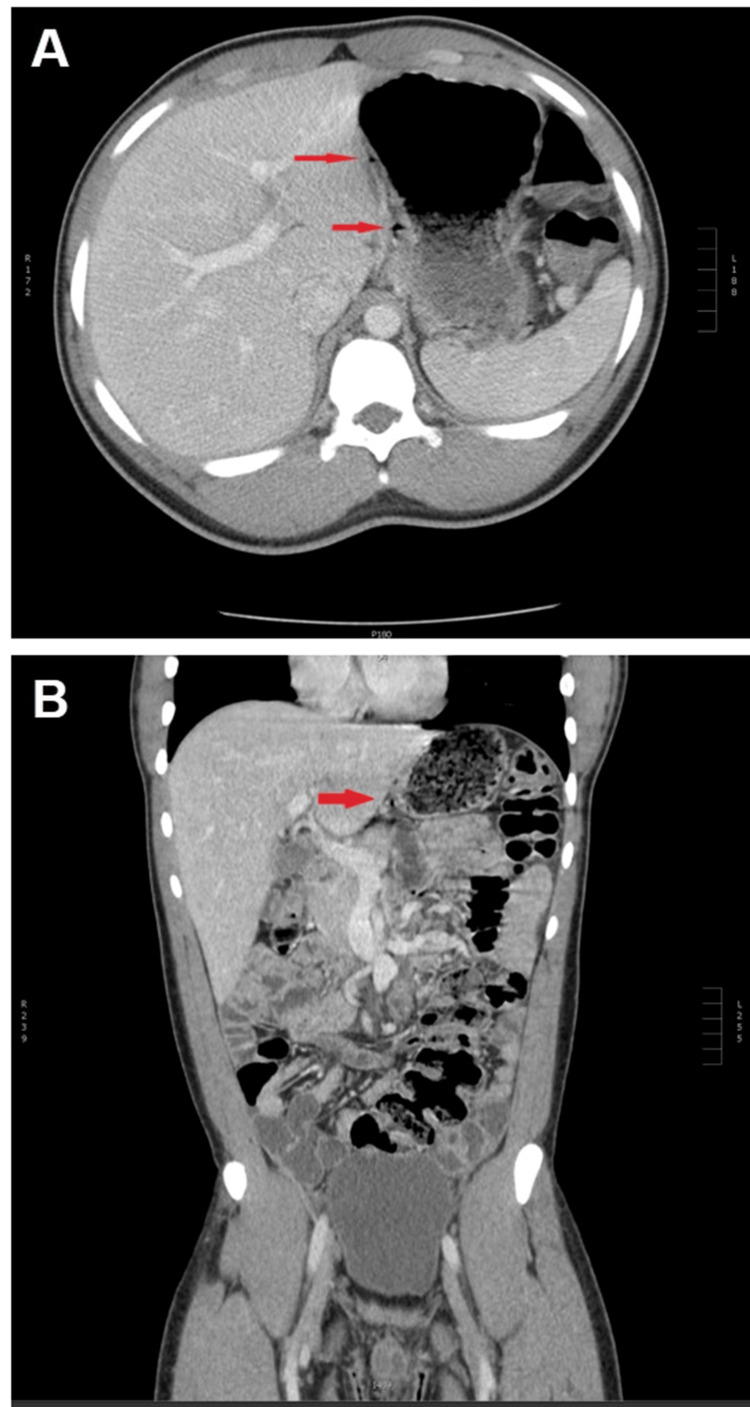
Images of the admission computed tomography scan with IV contrast of the abdomen/pelvis. (A) Axial and (B) coronal scans with arrows indicating tiny air density foci in the subcapsular region of the left lobe of the liver from the gastrohepatic ligament going medial to the wall of the stomach. This is a suboptimal view, as the CT scan was done in the portal venous phase instead of the preferred arterial phase. We believe this shows evidence of inflammation of the liver capsule despite not showing the characteristic liver capsule enhancement expected in an arterial phase scan.

An admission CT with IV contrast of the abdomen and pelvis showed tiny air density foci in the subcapsular region in the left lobe of the liver in the gastrohepatic ligament medial to the wall of the stomach and no free air or perforation (Figure 1). No appendix abnormalities were noted on the admission CT. Admission gallbladder ultrasound had no abnormalities. General surgery and infectious diseases were consulted, but no surgical intervention was performed given the lack of active infection indicators on labs or imaging. The patient developed a fever of 38.1°C on hospital day one and continued to experience pain out of proportion to the exam, requiring high doses of opioid analgesics. Magnetic resonance imaging of the liver with and without contrast, as well as magnetic resonance cholangiopancreatography, showed no further abnormalities.

The patient underwent testing for sexually transmitted infections, resulting in negative results for HIV, gonorrhea, and trichomonas but a positive result for *Chlamydia trachomatis* via urine PCR. At this time, inflammatory markers were also assessed, with results showing a normal erythrocyte sedimentation rate of 1 mm/hr and an elevated CRP level of 2.65 mg/dL, suggesting an inflammatory process that could be producing intra-abdominal adhesions. Admission blood cultures showed no growth. Diagnoses of appendicitis, cholecystitis, and bowel ischemia were eliminated based on laboratory and imaging. A diagnosis of chlamydia and perihepatitis was made clinically without a diagnostic laparoscopy to confirm the diagnosis and save the patient from an invasive procedure. The patient was discharged on hospital day three with a prescription for doxycycline 100 mg twice daily for seven days.

Following discharge, the patient had several emergency department visits for abdominal pain at an outside hospital system and, notably, developed a partial small bowel obstruction 13 months following this admission. It is unknown if the patient completed his full course of antibiotics or if he had sufficient primary care follow-up, although primary care follow-up is doubtful given how many subsequent times the patient visited the emergency department. 

## Discussion

Perihepatitis is likely underdiagnosed due to its rarity, especially in male patients. In patients with right upper quadrant pain in the absence of gallstones or other obvious intra-abdominal processes, perihepatitis should be considered in the differential diagnosis. A dedicated CT scan using arterial phase for contrast may be needed to visualize liver capsule enhancement, as delayed phase imaging has a substantially lower sensitivity for liver capsule enhancement. This may have contributed to the lack of characteristic liver capsule enhancement in our patient. A detailed sexual history should be taken, and testing for gonorrhea and chlamydia infection should be performed.

Contrast-enhanced computed tomography (CT) scan of the abdomen is the preferred initial imaging modality to diagnose perihepatitis and generally shows liver capsule enhancement [4]. One study showed that arterial phase CT always showed liver capsule enhancement in patients with perihepatitis, but portal phase CT showed enhancement in only 62% of cases [4]. Perihepatitis is commonly diagnosed by a positive test for chlamydia or gonorrhea, accompanied by right upper quadrant pain and suggestive findings on an arterial phase CT scan [2,6].

**Figure 2:**
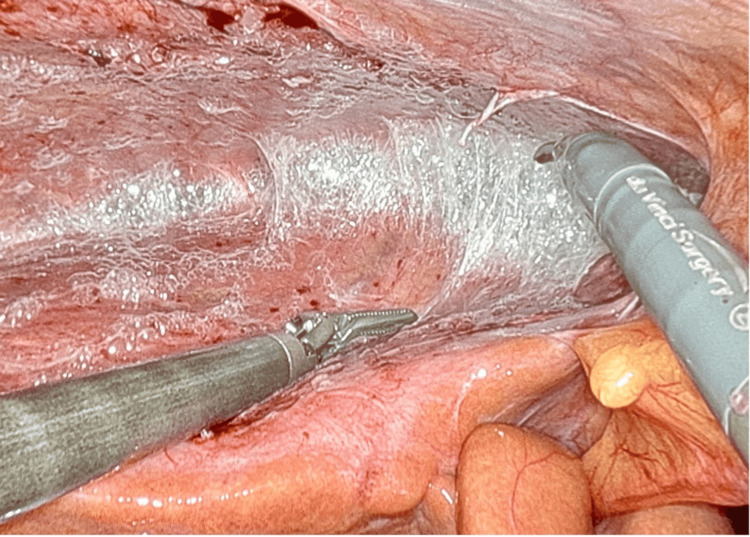
Example of violin string-like adhesions seen on laparoscopy. Source [3]

Perihepatitis has two stages: the acute stage, characterized by mild exudative inflammation of the liver capsule, and the chronic stage, marked by the formation of adhesions [4,7]. The characteristic violin string-like adhesions are shown in Figure 2. Patients in the acute phase often present with fever, leukocytosis, elevated C-reactive protein (CRP) levels, and episodic right upper quadrant abdominal pain [3]. Patients in the chronic phase often present with dull right upper quadrant abdominal pain [3]. During laparoscopy, the severity of the disease can be classified as mild (≤5 adhesions between the right lobe of the liver and the abdominal wall), moderate (≥6 adhesions), or severe (involvement of both liver lobes) [8]. There is no available data on the sensitivity and specificity of a diagnosis based on clinical history, laboratory data, and imaging alone compared to laparoscopy, likely due to the very few numbers of reported male cases of perihepatitis.

The mechanism by which perihepatitis develops from an ascending genitourinary infection is not fully understood [5,7]. In females, an ascending infection putatively exits the fallopian tubes into the abdominal cavity [3,5,7]. Because this pathway does not exist in males, the suspected mechanism is hematogenous or lymphatic spread [4,5,7]. Antibiotic treatment targeting the causative organism is usually sufficient to resolve the disease [1]. In some cases, patients may experience long-term complications, including chronic pain, small bowel obstruction due to adhesions, and infertility in women [6]. Adhesions can also complicate subsequent intra-abdominal surgeries, such as laparoscopic cholecystectomy [9]. Patients may undergo laparoscopic lysis of adhesions if adhesions are found during laparoscopy, if chronic pain persists despite an appropriate course of antibiotics, or if a small bowel obstruction develops [3].

Epidemiologically, there are extremely limited numbers of reported male cases of perihepatitis. The first reported male case by Lieutenants Kimball and Knee was in 1970, despite female cases being reported in the 1930s [3,10]. A table of known case reports of male perihepatitis is shown in Table 1. Given this case report will only be the eighteenth case known to us, it appears that this condition is often unrecognized or underreported.

**Table 1:** List of known case reports of male patients with perihepatitis All treatments with laparoscopy included lysis of adhesions as indicated.

Authors	Year of Publication	Patient age (years)	Pathogen Isolated	Specimen Type	Treatment
Kimball et al. [10,11]	1970	22	N. gonorrhoeae	Liver tissue	Penicillin
Francis et al. [11]	1972	25	N. gonorrhoeae	Urine culture	Penicillin
Fung et al. [11]	1981	26	N. gonorrhoeae	Rectal swab	Penicillin, ampicillin
Davidson et al. [11]	1982	35	N. gonorrhoeae	Pharyngeal swab	Penicillin
Winkler et al. [11]	1985	35	N. gonorrhoeae	Pharyngeal swab	Penicillin, amoxicillin
Baek et al. [11]	2010	35	M. genitalium	Urine PCR	Levofloxacin, doxycycline
Saurabh et al. [11]	2012	29	None isolated	Ascites culture	Laparoscopy
Rouhard et al. [11]	2014	45	None isolated	Ascites culture, urine culture	Quinolone, metronidazole
Jeong et al. [4,11]	2015	24	E. faecalis	Urine culture	Levofloxacin, doxycycline
Yi et al. [6,11]	2015	60	None isolated	Urine culture, urine PCR	Levofloxacin
Nardini et al. [11]	2015	26	N. gonorrhoeae	Urine PCR	Ceftriaxone, azithromycin
Takata et al. [11]	2018	50	C. trachomatis	Urine PCR	Levofloxacin
Lisičar et al. [2]	2019	33	C. trachomatis	Rectal swab	Ceftriaxone, doxycycline
Coco et al. [12]	2022	40	None isolated	None taken	Laparoscopy
Azrielant et al. [13]	2023	37	C. trachomatis	Ascites culture	Laparoscopy, doxycycline
Niang et al. [14]	2024	58	C. trachomatis	Urine culture	Doxycycline, ceftriaxone, isoniazid, rifampicin, pyrazinamide, ethambutol
Mostafa et al. [3]	2024	33	None isolated	None taken	Laparoscopy
Our case	2025	18	C. trachomatis	Urine PCR	Doxycycline

## Conclusions

A missed diagnosis may increase the risk of long-term complications from perihepatitis, such as our patient who ultimately suffered from chronic abdominal pain and small bowel obstruction, and unknowingly spreading the infection to other sexual partners. Patients with chronic symptoms, such as our patients, may benefit from diagnostic laparoscopy. We recommend that sexually transmitted infection testing should be considered in males with unexplained abdominal pain, given PID is often only seriously considered in female patients. Early recognition and treatment can reduce the formation of intra-abdominal adhesions and thus reduce the likelihood of chronic symptoms. While a presumptive diagnosis can be made with a combination of clinical appearance, imaging, laboratory data, and exclusion of other causes, as we have done for this case, a true diagnosis needs laparoscopy. Laparoscopy is often not done due to invasiveness but should be considered if empirical treatment from a presumptive diagnosis does not resolve symptoms. For all patients with suspected or confirmed pelvic inflammatory disease, we recommend close outpatient follow-up with primary care to assure completion of antibiotic treatment, resolution of symptoms, and, if still symptomatic, arrangement of further evaluation. Recognition of perihepatitis in males is currently poor given the small number of case reports in the literature and can be enhanced by emphasizing in medical education that perihepatitis does also occur in males. Prevention of perihepatitis can be enhanced by better educating the general public about the benefits of regular and frequent testing for sexually transmitted infections, leading to treatment before intraabdominal infections develop. We hope the addition of our case report to the literature aids in improving the recognition of this condition.
